# Transcriptomics and disease vector control

**DOI:** 10.1186/1741-7007-8-52

**Published:** 2010-05-07

**Authors:** John Vontas, Hilary Ranson, Luke Alphey

**Affiliations:** 1 Laboratory of Molecular Entomology, Faculty of Biotechnology and Applied Biology, Department of Biology, University of Crete, Heraklio, 71409, Greece; 2 Vector Group, Liverpool School of Tropical Medicine, Liverpool, L35QA, UK; 3 Oxitec Ltd, 71 Milton Park, Oxford OX14 4RX, UK; 4 Department of Zoology, University of Oxford, South Parks Road, Oxford OX1 2PS, UK

## Abstract

Next-generation sequencing can be used to compare transcriptomes under different conditions. A study in *BMC Genomics *applies this approach to investigating the effects of exposure to a range of xenobiotics on changes in gene expression in the larvae of *Aedes aegypti*, the mosquito vector of dengue fever.

See research article http://www.biomedcentral.com/1471-2164/11/216

## Commentary

Prevention of malaria and other mosquito-borne diseases depends in large part on vector control and usually involves the use of insecticides. Insecticide-based methods include insecticide-impregnated bed nets, as well as more obvious applications such as indoor or aerial sprays and water treatments. However, the emergence and spread of insecticide resistance poses a serious threat to the sustainability of current control efforts [1], with several insecticide classes already showing reduced efficacy in controlling disease vectors in the field. Thus, there is a clear and urgent need for improving the sustainability of current insecticide-based control interventions as well as for exploring alternative, non-insecticidal methods for controlling major vectors.

Identification of the factors influencing the selection of insecticide resistance is an important prerequisite for managing insecticide resistance. A recent study in *BMC Genomics *by David *et al. *[2] employs next-generation sequencing methods to analyze the transcriptional response of the mosquito *Aedes aegypti*, the vector of the dengue virus, to xenobiotics (manmade chemicals) potentially present in its aquatic habitat. Studies such as this may ultimately lead to novel strategies to overcome resistance in the field.

## Insecticide-resistance mechanisms

Insecticide resistance is typically characterized by a variety of molecular aberrations, such as transcriptional changes, gene amplification and point mutations in coding regions, which result in increased rates of insecticide detoxification or reduced sensitivity of the target protein(s). Analysis of mRNA has provided significant insights into the molecular basis of resistance. Over the years, research in the field has progressed from analysis of a small number of candidate genes to high-throughput expression profiling driven by the advent of microarrays.

Specific microarray platforms, known as Detox Chips, have been developed for the analysis of resistance mechanisms in mosquitoes [3]. These targeted microarray platforms and whole-genome arrays have identified a number of candidate insecticide-resistance genes. These include detoxification enzymes [3], some of which have been confirmed to metabolize insecticides [4] and have become targets for the design of inhibitors aimed at inactivating insecticide-metabolizing enzymes in natural populations. Several other genes, such as those involved in formation of the insect cuticle and those involved in the mitochondrial respiratory chain, have also been associated with the resistance phenotype [3-5] but a causal role has not yet been demonstrated in mosquitoes.

An important limitation of microarray technology for studying insecticide resistance is its inability to identify mutations in detoxification enzymes that may confer resistance by altering the metabolic efficiency of the enzyme for insecticides. Although only a few cases have been documented in the literature (reviewed in [6]), Chiu *et al. *[7] have shown that P450 cytochromes that are very similar in sequence can have dramatically different insecticide-metabolism profiles, and the authors suggest that a systematic analysis for allelic variants is essential. The advent of next-generation sequencing provides new opportunities for transcriptomic studies of insecticide-resistance mechanisms. Transcripts from susceptible and resistant insects can be compared to identify sequence polymorphisms and, provided sufficient sequence depth is achieved, quantitative data on transcript levels in the different populations can be obtained.

The importance of exposure to sublethal concentrations of insecticides in inducing resistance remains controversial. Most conventional insecticides act very rapidly and it is unlikely that the induction of detoxification enzymes will play an important part in determining the fate of the insect upon exposure to a pyrethroid-treated net. However, exposure to sublethal concentrations of insecticides or other xenobiotics in the larval habitats undoubtedly plays an important part in shaping the larval, and perhaps the adult, mosquitoes' tolerance to insecticides. To investigate this response, David *et al. *[2] sequenced the entire transcriptome of *Ae. aegypti *larvae from populations exposed to different xenobiotics during their development. They showed that a large number of genes (including those encoding transporters, and enzymes involved in the mitochondrial respiratory chain and detoxification processes) were affected in a general response - defined as 'all proteins over-produced due to environmental stress' [2] - to some xenobiotics (such as the insecticides propoxur and fluoranthene) and in a more specific response (smaller number of affected genes) to others (permethrin, atrazine and copper). The authors suggest that their findings indicate a 'hidden impact' of anthropogenic pollutants on ecosystems with possible consequences and practical implications for vector control.

## Next-generation sequencing technologies in disease vector control research

**Figure 1:**
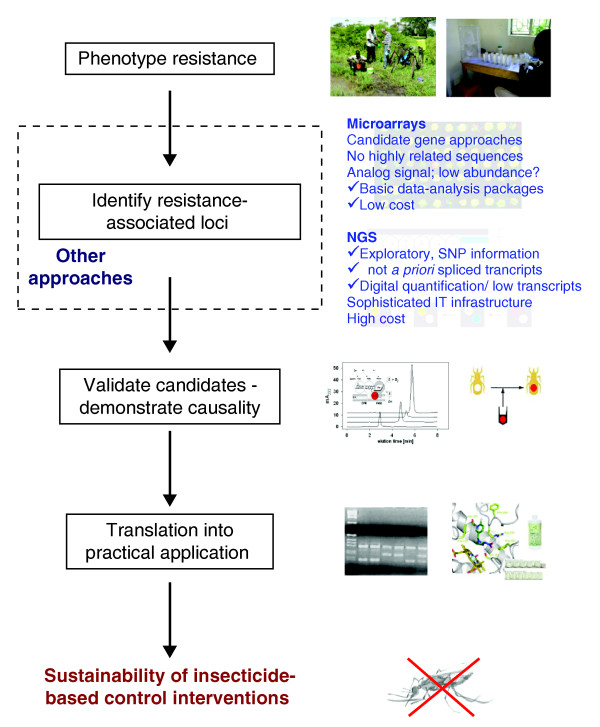
**The contribution of transcriptional profiling approaches in insecticide-resistance research for improving the sustainability of insecticide-based disease vector control interventions**. NGS, next-generation sequencing; SNP, single-nucleotide polymorphism.

Next-generation sequencing has revolutionized transcriptome analysis by providing genome-wide expression profiles that are both quantitative and precise, affording single-nucleotide resolution of differences between sequences [8]. In essence, RNA (converted into cDNA) is sequenced in massively parallel sequencing instruments such that the number of times a sequence is represented in the output sequence data represents its relative abundance in the input RNA - in other words its expression level. Transcriptome analysis by this method has some significant advantages over microarray platforms (Figure 1), including analysis of polymorphisms in the coding sequence and the ability to detect novel transcripts. David *et al. *[2] identified a large number of sequence clusters not located within predicted genes in the *Ae. aegypti *genome, which possibly indicate novel alternative splice junctions and transcriptional units. However, accurate estimates of low-abundance transcripts and the analysis of exon polymorphism become apparent only if sequencing depth is sufficient, and this requirement dramatically increases the cost of the sequencing. In addition, a sophisticated information-technology infrastructure is required. The cost and data-processing requirements make it unlikely that next-generation sequencing will replace microarrays as the routine tool for expression profiling in disease vector control applications in the short term, even in a research context.

Transcriptomics has applications to vector control that extend beyond understanding and managing insecticide resistance. One is in the area of genetic control strategies - the use of genetically engineered mosquitoes to reduce disease transmission. There are several types of approaches to this [9], but all require exogenous sequences to be expressed at a predetermined time, place and/or level in the mosquito. But although there are a large number of such times, places and levels of interest to mosquito genetic engineers, there are very few characterized promoters and other control elements with the necessary specificity. To develop a strain for a 'sterile-male' genetic control system [10], Fu *et al. *[11] used sex-specific alternative splicing, and the 'tet-off' gene-expression system, in combination with a promoter identified via subtractive hybridization of cDNAs. This was used to give sex- and tissue-specific expression of an effector molecule and, by design, a phenotype of repressible female-specific flightlessness. Next-generation sequencing could greatly facilitate the identification of suitable transcripts leading to potentially useful promoters, and also of alternative splicing systems. More generally, the potential value of systematic analysis is clear from ongoing work in *Drosophila melanogaster*. Release FB2010_03 of FlyBase [12] includes genome-wide transcription profiles at multiple developmental stages. Expression profiles, transcription start and stop sites, and alternative splicing patterns can all be inferred from these data. Furthermore, even though *Drosophila *already had one of the best annotated of all metazoan genomes, transcriptomics based on next-generation sequencing has revealed numerous new or incorrectly annotated transcription units.

Although next-generation sequencing might improve the quality of transcriptome profiling in the medium term, switching to novel technologies should not be seen as a magic bullet, however. Progress in vector-control research is just as often hindered by inappropriate study design and data interpretation as by technical issues. For example, differential gene expression between insecticide-resistant and susceptible mosquitoes may reflect different geographical origins and/or genetic background, and not be related to the resistance phenotype. The new sequencing technologies are clearly a great addition to the available tools in vector-control research. The challenge will be how to use them to generate biologically meaningful data and clear interpretations to drive follow-up experimentation, rather than producing piles of data as an alternative to thinking. The full potential of sequencing-based transcriptomics will be seen when it is used sensibly in combination with additional functional genomics approaches to validate predictions that arise from these datasets.
